# Seasonality of Tuberculosis in Delhi, India: A Time Series Analysis

**DOI:** 10.1155/2014/514093

**Published:** 2014-03-23

**Authors:** Varun Kumar, Abhay Singh, Mrinmoy Adhikary, Shailaja Daral, Anita Khokhar, Saudan Singh

**Affiliations:** Department of Community Medicine, Vardhman Mahavir Medical College and Safdarjung Hospital, New Delhi 110029, India

## Abstract

*Background*. It is highly cost effective to detect a seasonal trend in tuberculosis in order to optimize disease control and intervention. Although seasonal variation of tuberculosis has been reported from different parts of the world, no definite and consistent pattern has been observed. Therefore, the study was designed to find the seasonal variation of tuberculosis in Delhi, India. *Methods*. Retrospective record based study was undertaken in a Directly Observed Treatment Short course (DOTS) centre located in the south district of Delhi. Six-year data from January 2007 to December 2012 was analyzed. Expert modeler of SPSS ver. 21 software was used to fit the best suitable model for the time series data. *Results*. Autocorrelation function (ACF) and partial autocorrelation function (PACF) at lag 12 show significant peak suggesting seasonal component of the TB series. Seasonal adjusted factor (SAF) showed peak seasonal variation from March to May. Univariate model by expert modeler in the SPSS showed that Winter's multiplicative model could best predict the time series data with 69.8% variability. The forecast shows declining trend with seasonality. *Conclusion*. A seasonal pattern and declining trend with variable amplitudes of fluctuation were observed in the incidence of tuberculosis.

## 1. Introduction

Tuberculosis (TB) which can be disseminated widely from active cases through aerosol droplets is an often fatal infectious disease caused by the agent* Mycobacterium tuberculosis*. Despite many efforts to control this disease, TB remains a major public health issue with a high global health burden, particularly with the emergence of multidrug resistant (MDR), extensive drug resistant (XDR), and the recent emergence of total drug resistant (TDR) strains along with coinfection with human immunodeficiency virus (HIV) especially in developing countries [1]. Each year, an estimated 9 million new cases of tuberculosis occur worldwide and responsible for an estimated 1.7 million deaths [2].

While seasonal variation has been widely reported for many respiratory infections in different parts of the world, it is much less documented for TB. There is paucity of data on seasonal variation in pulmonary TB in developing countries, although a number of studies in developed countries have reported peaks in late winter and early spring or summer [3, 4]. Thorpe et al. have found that in Northern India, TB diagnosis peaked between April and June and it reached a nadir between October and December, but in Southern India, no such seasonal variation was found [5].

India bears a disproportionately large burden of the world's tuberculosis rates, as World Health Organization (WHO) statistics for 2011 giving an estimated incidence figure of 2.2 million cases of TB for India out of a global incidence of 8.7 million cases [6]. Since 1998, effective tuberculosis control programs have been rapidly expanding across India allowing for population based analyses with standardized register data.

A study on the economic impact of scaling up of Revised National Tuberculosis Control Program (RNTCP) in India in 2009 shows that on an average each TB case incurs an economic burden of around US$ 12,235 and a health burden of around 4.1 disability adjusted life years (DALYs). Similarly, a death from TB in India incurs an average burden of around US$ 67,305 and around 21.3 DALYs [7]. So the present study was conducted to find if there is any seasonal variability of TB incidence in Delhi, India, and to create the best possible univariate model for TB monthly incidence for the last six years with the available time series data.

## 2. Materials and Methods

The present study was a retrospective record based study undertaken in a Directly Observed Treatment Short course (DOTS) centre of Fatehpur Beri primary health centre located in the south district of Delhi, which caters to a population of 64,000. Three sputum smear examinations were done for acid-fast bacilli (AFB) as per the guidelines of RNTCP. Six-year data from January 2007 to December 2012 were analyzed. The cases were assigned to the month of first positive smear and a total of 417 new smear positive cases of TB were registered during the study period.

Expert modeler of SPSS ver.21 software was used to fit the best suitable model for the time series data. The stationarity of the data was checked by autocorrelation function (ACF) and partial autocorrelation function (PACF). Seasonal adjusted factor (SAF) was used to determine the peak of seasonal variation. The Ljung-Box (modified Box-Pierce) test was used to determine if the model was correctly specified. Forecasting of the incidence of monthly TB cases was also done using the best fit model.

## 3. Results

According to RNTCP guidelines, 18 (4.3%) were pediatric TB cases and 399 (95.7%) were adults (Table 1).

**Figure 1:**
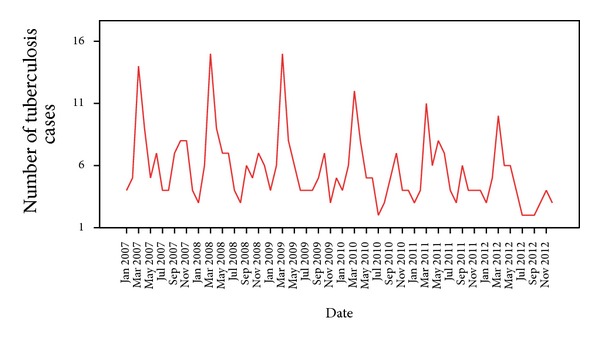
Sequence chart of total number of tuberculosis.

Figure 1 clearly shows that there has been a steady declining trend in the TB incidence over the study period. The series exhibited a number of peaks; aside from the small scale fluctuations, the significant peak appeared to be separated by more than a few months. It shows a cyclical seasonal pattern as the peak of newly registered cases follows a similar pattern with an interval of few months between the peaks.  Table 2 shows the monthly TB incidence during the study period from January 2007 to December 2012.

**Figure 2:**
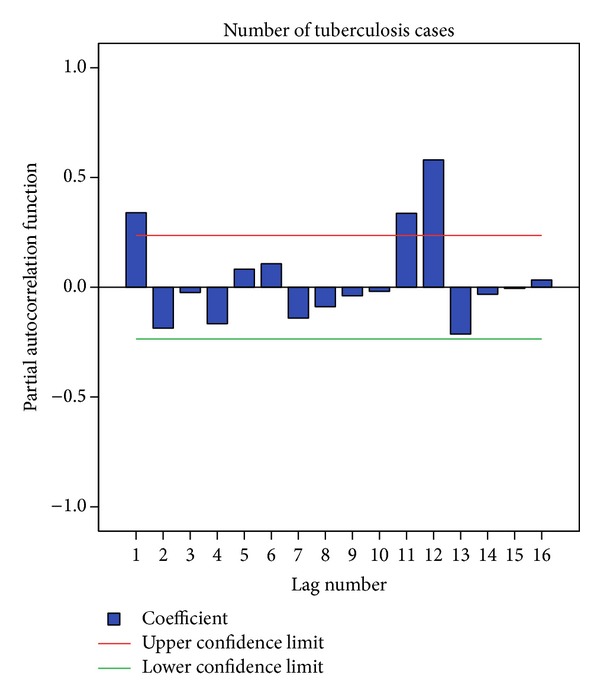
Partial autocorrelation plot for tuberculosis cases.

The autocorrelation function showed a significant peak at a lag of 12 (autocorrelation = 0.698; Box-Ljung statistics (*P* = 0.000)) suggesting the presence of a seasonal component in the data. Auto correlation function (ACF) showed a significant peak at a lag of 12. Partial autocorrelation function (PACF) also showed a significant peak at a lag of 12 which confirmed the presence of a seasonal component in the data (Figure 2).

**Figure 3:**
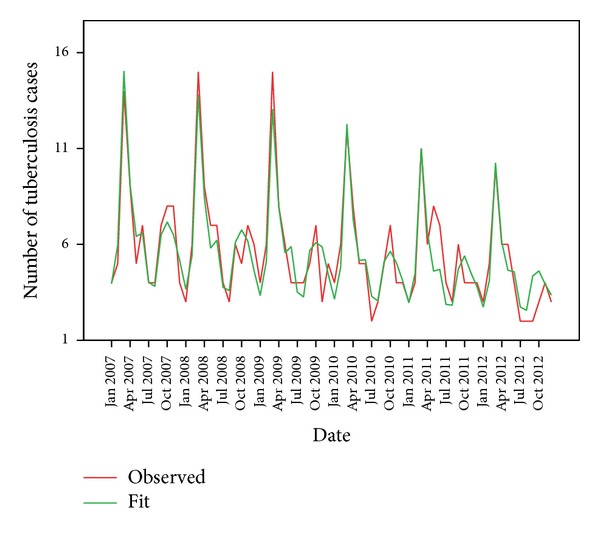
Actual (observed) and predicted (fit) values of tuberculosis cases.

The time series sequence chart (Figure 1) shows both declining trend and periodic seasonal fluctuations. So the expert modeler of SPSS ver. 21 suggested Winter's multiplicative model as the best fitted mathematical model for this time series data.  Figure 3 shows that the observed actual values and the predicted model values matched reasonably well and there was consistency in the trend.

The Ljung-Box (modified Box-Pierce) test indicated that the model was correctly specified (Table 3). The expert modeler detected no outliers in the data.

Although the time series modeler offers a number of different goodness of fit statistics, here stationary *R*-squared value was used. This statistic provides an estimate of the proportion of the total variation in the series that is explained by the model and is preferable to ordinary *R*-squared when there is a trend or seasonal pattern, as is the case here. Larger values of stationary *R*-squared (up to a maximum value of 1) indicate better fit. A value of 0.698 meant that the model could explain 69.8% of the observed variation in the series.

In Winter's multiplicative model, the seasonal adjustments are multiplied by the seasonally adjusted series to obtain the observed values. This adjustment attempts to remove the seasonal effect from a series in order to look at other characteristics of interest that may be “masked” by the seasonal component in effect seasonal components that do not depend on the overall level of the series. Observations without seasonal variation have a seasonal component of 0.  Table 4 shows that from the month of March to June the seasonal adjusted factor (SAF) of TB was more than 0; that is, in these months the registered TB cases were more above the typical months. Among these months, March with SAF = 216.4 had the highest SAF; that is, in this month, the registered TB cases were more than 216.4% above the typical months.

The months of March, April, May, and October have SAF of more than 100. This shows that during these months the number of newly registered TB cases was more than the typical months. This shows that the peak of new TB cases occurs during these months which coincides with the spring season in Delhi.

**Figure 4:**
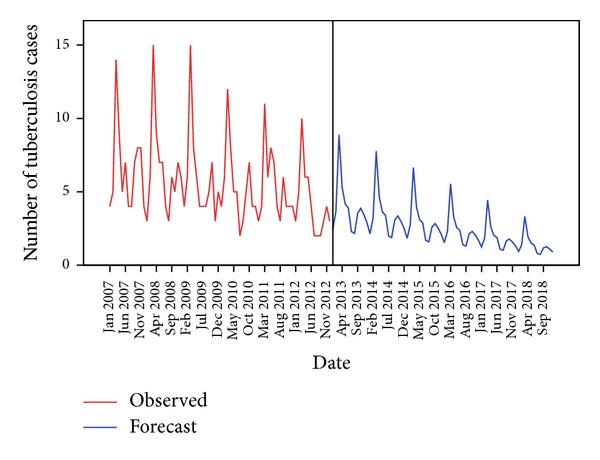
Sequence chart for forecasted tuberculosis cases in the near future.

The same model was used to forecast the monthly tuberculosis incidence for the future from January 2013 to December 2018 and the values are depicted in Figure 4.

The forecasted values also show a declining trend over the years with a peak during the month of March.

## 4. Discussion

In a previous study conducted in India on assessment of seasonal trends, Thorpe et al. [5] reported that diagnosis of TB peaked between April and June and reached nadir between October and December. Areas in the north India had the highest seasonal variation and low or no seasonality was noted in central and southern regions of India in that study. Seasonal variations have also been reported from various other countries like China [8] and United Kingdom [9]. Since seasons involve variations in various phenomena like temperature, humidity, precipitation, length of daylight, and so forth and also vary by geography and latitude, the presence of seasonal variation and the timing and magnitude of such seasonal variation may depend on some of these factors in ways not yet fully understood. Apart from these reasons, differential access to health care with varying seasons can also be an important factor in new TB case detection. But in the present study, this factor plays a very little role.

The real causes of seasonal patterns of TB remain unknown, but the seasonal trend, with higher incidence rate in winter, may be relevant to the increased periods spent in overcrowded, poorly ventilated housing conditions, these phenomena much more easily seen in winter than during warm seasons. The outside environment determines the amount of time spent indoors and thus the transmissibility of* Mycobacterium tuberculosis* [4]. In the present study, indoor air pollution seems to be a plausible cause for this striking and sustained seasonal pattern in the number of TB cases which can be either primary or reactivated.

A possible link between vitamin D deficiency and impaired host defense to* Mycobacterium tuberculosis* infection leading to primary TB has also been postulated [10]. The relation between exposure to sunlight and risk for active tuberculosis has been increasingly recognized, with the hypothesis being vitamin D deficiency reducing the ability of macrophages to kill intracellular* Mycobacterium tuberculosis* [11]. Delhi is situated in the subtropical region at a latitude level of around 28.2 degree north of equator and plenty of sunlight is expected all-year round except during winter months of December and January.

Another reason for the onset of adult tuberculosis is thought to be due to reactivation, although the mechanism of reactivation and development of overt tuberculosis is not well understood, but it has been attributed to poor nutrition and socioeconomic status [12, 13]. Seasonal changes in the absolute numbers and ratios of T helper and suppressor cells could possibly alter cell mediated immunity, which is crucial to the host response controlling infection with* Mycobacterium tuberculosis*. However, the factors that regulate the seasonal changes in T cell subset numbers or function remain unknown [14].

Based upon the results of this study, we believe that there will be no obvious improvement in the high burden of TB in India in the near future. The results indicate that in the near future, the reported annual TB incidence numbers in India will decrease only slightly. The results of the study can be used for appropriate allocation of resources to target prevention and treatment of tuberculosis. It can also be used to inform travelers on TB risk and screening. Another potential implication of this study's findings are that, given the seasonal variation observed, clinicians, even in locations other than India, should have a higher than usual clinical suspicion for TB (and perhaps a lower threshold to place patients in airborne isolation) in the late winter and spring months.

But the study has its own limitations, with the major one being the lack of clinical data. Since the study is of observational design, the cause and effect relationship could not be established. It is also to be noted that it is a DOTS centre record based study and more sick people are likely to attend tertiary hospital. So it is difficult to predict the variation in the actual population.

## 5. Conclusion

A seasonal pattern of TB was observed for newly diagnosed smear positive cases with variable amplitudes of fluctuation. These observations are suggestive of the presence of a seasonal disease-modifying factor. This regularity of peak seasonality in TB case detection may prove useful to initiate measures that warrant a better implementation of control measures. This information would be also useful for administration and managers to take extra care to arrange and provide extra facilities during the peak seasons.

## Figures and Tables

**Table 1:** Distribution of study subjects according to age and sex (*n* = 417).

Gender	Age (in years)		Total
	≤14	≥15	
Male	11 (2.6)	273 (65.5)	284 (68.1)
Female	7 (1.7)	126 (30.2)	133 (31.9)

Total	18 (4.3)	399 (95.7)	417 (100)

**Table 2:** Monthly tuberculosis incidence during the study period (*n* = 417).

Month	2007	2008	2009	2010	2011	2012
January	4	3	4	4	3	3
February	5	6	6	6	4	5
March	14	15	15	12	11	10
April	9	9	8	8	6	6
May	5	7	6	5	8	6
June	7	7	4	5	7	4
July	4	4	4	2	4	2
August	4	3	4	3	3	2
September	7	6	5	5	6	2
October	8	5	7	7	4	3
November	8	7	3	4	4	4
December	4	6	5	4	4	3

Total	89	78	71	65	64	50

**Table 3:** Model statistics for tuberculosis data.

Model parameter	Stationary *R* ^2^	Ljung-Box statistic			Model type
		Statistics	df	*P* value	
Tuberculosis monthly incidence	0.698	17.88	15	0.269	Winter's multiplicative model

**Table 4:** Seasonal adjustment factor (SAF) for tuberculosis cases.

Month	Observed cases	SAF (%)
January	21	59.9
February	32	95.5
March	77	216.4
April	46	132.6
May	37	113.5
June	34	97.2
July	20	58.7
August	19	57.2
September	31	96.8
October	34	105.1
November	30	86.8
December	26	80.4
